# Anticoagulation Management Post Pulmonary Embolism

**DOI:** 10.14797/mdcvj.1338

**Published:** 2024-05-16

**Authors:** Joseph J. Naoum

**Affiliations:** 1 Houston Methodist Hospital Clear Lake, Nassau Bay, Texas, US; 2 Houston Methodist DeBakey Heart & Vascular Center, Houston, Texas, US

**Keywords:** venous thromboembolism, anticoagulation, deep vein thrombosis, pulmonary embolus, revascularization, unfractionated heparin, low molecular weight heparin, direct oral anticoagulants, venotonics

## Abstract

Pulmonary embolus (PE) carries a significant impending morbidity and mortality, especially in intermediate and high-risk patients, and the choice of initial anticoagulation that allows for therapeutic adjustment or manipulation is important. The preferred choice of anticoagulation management includes direct oral anticoagulants. Vitamin K antagonists and low-molecular-weight heparin are preferred in special populations or selected patients such as breastfeeding mothers, those with end-stage renal disease, or obese patients, among others. This article reviews the primary and longer-term considerations for anticoagulation management in patients with PE and highlights special patient populations and risk factor considerations.

## Introduction

Venous thromboembolism (VTE) refers to the occurrence of a deep vein thrombosis (DVT) or pulmonary embolus (PE). The latter carries a significant impending morbidity and mortality. For low-risk patients, a regimen of anticoagulation in the outpatient setting can be started. For intermediate and high-risk patients who are scheduled for pulmonary revascularization or who may need it pending clinical deterioration, the choice of initial anticoagulation that allows for therapeutic adjustment or manipulation is important. This article reviews the primary and longer-term considerations for anticoagulation management in patients with PE and highlights special patient populations and risk factor considerations.

## Initial Choice of Anticoagulation

Traditionally, the anticoagulant treatment for patients with venous thrombosis involved the initiation of an unfractionated heparin (UFH) drip with transition to vitamin K antagonists (VKA). Later, low-molecular-weight heparin (LMWH) subcutaneous injections eased the transition to VKAs. This approach often required prolonged hospitalization and monitoring of VKA therapeutic levels.

This approach was replaced starting in 2010 with the introduction of direct oral anticoagulants (DOAC), which do not require therapeutic level monitoring and provide a standard dosing requirement specific to the choice of medication. Only dabigatran and edoxaban need to be preceded with a course of parental anticoagulation with either UFH or LMWH. Rivaroxaban and apixaban can be started directly, making them some of the most prescribed DOACs in this class.^1^ Furthermore, DOACs have been associated with decreased risk of bleeding compared to VKAs, and societal guidelines now favor their use for initial and long-term treatment. This therapeutic shift has contributed to shortened length of hospital stay (LOS) and, in certain low-risk cases, avoidance of hospitalization altogether.^2,3^

While therapeutic anticoagulation is the standard treatment for PE, some patients with intermediate to high-risk PE may require open surgical or catheter-related intervention, and the choice of initial anticoagulant may change pending intervention. Unfractionated heparin or LMWH may initially be preferred for patients who may require reperfusion therapy, after which long-term standard anticoagulation can be instituted. In patients who have been started on UFH, less than 50% achieved stable therapeutic levels within the first 24 hours of initiation.^4^ Alternatively, with LMWH, predictable levels of anticoagulation can be achieved early after administration. As a result, LMWH is favored by many as the initial anticoagulant in at-risk patients in whom catheter-directed therapy is considered and for reinitiating anticoagulation immediately following intervention. Subsequently, patients can be transitioned to the choice of outpatient therapeutic anticoagulation, usually DOACs. Finally, low-risk PE patients can be readily started on DOACs as a first-line therapy during the initial encounter.^4, 5, 6^

Anticoagulation helps prevent the propagation of thrombus, avoid recurrence, and allow for the body’s intrinsic fibrinolytic activity or recanalization to take place over time. However, there are certain populations in which the choice of anticoagulation needs special attention.

## Special Populations

### Pregnancy and Breastfeeding

Women with PE who are pregnant or breastfeeding require UFH or LMWH since they do not cross the placenta or absorb into breast milk.^7, 8, 9, 10^ Alternatively, fondaparinux may have minor transplacental passage and can be considered when there is an allergy or adverse response to LMWH. DOACs and VKAs cross the placenta, can be teratogenic, and can cause fetal hemorrhage and placental abruption. Also, VKAs can cause central nervous system anomalies during pregnancy.^10, 11^ DOACs are contraindicated in patients who are breastfeeding,^7,11,12^ while LMWH and VKA can be given to breastfeeding mothers.^11^ Table 1 depicts anticoagulation recommendations during the pre- and postpartum phase of pregnancy.

**Table 1:** Anticoagulation recommendations during pregnancy in the pre- and postpartum phase. UFH: unfractionated heparin; LMWH: low-molecular-weight heparin; VKA: vitamin K agonists; DOAC: direct oral anticoagulant


PRE- AND POST-PARTUM ANTICOAGULATION TREATMENT TIMELINE IN PE	

DURATION	CHOICE OF ANTICOAGULANT

**Acute**	UFH

0-days	LMWH*

**Standard Pre-partum**	LMWH*

Post-partum **	

6 weeks – 3 months	LMWH

* Breast feeding*	VKA

* Not breast feeding*	LMWH

	VKA

	DOAC


*Fondaparinux can replace low-molecular-weight heparin when contraindicated. **Continuation of therapy should consider persistent risk factors and timelines as depicted in Table 2.

### Kidney Disease

VKAs have been the maintenance anticoagulant of choice for patients with end-stage renal disease (ESRD).^7^ DOACs show an increased total systemic plasma concentration exposure over time with decreasing renal function. In addition, drug half-life is at least doubled in patients with renal disease. This class of medication also appears to be poorly dialyzable.^9^ DOACs have been used in patients with renal disease at a reduced dose.

### Antiphospholipid Syndrome

Patients with confirmed antiphospholipid syndrome (APS) should have their maintenance treatment phase with VKA over DOACs.^5^ Studies comparing rivaroxaban with VKAs showed an increased thrombotic event with the former, although trials showed an increase in arterial rather than venous thromboses.^13^ Of interest, pooled data from trials using dabigatran did not show a difference in the incidence of venous thromboembolisms and deaths from using dabigatran versus warfarin, while bleeding events of any kind were less frequent with dabigatran use.^14^ In a review of the use of DOACs in patients with APS, the rate of VTE was similar between rivaroxaban and VKA, suggesting that rivaroxaban could be a safe alternative in APS. However, the authors further advise against implementing DOAC use in triple-positive APS patients and recommend VKAs instead for this at-risk group.^7,15^

### Obesity

For the treatment of acute VTE, weight should not be a significant factor in deciding which anticoagulant to use.^16^ Furthermore, DOACs can be considered in all obese patients regardless of body mass index (BMI). However, data is limited for excessive obesity (BMI > 50 kg/m^2^). There appears to be a similar risk of recurrence when comparing use of DOACs to VKA.^16^ Obese patients may require high doses of VKA and more time to achieve therapeutic levels.^9^ Capped doses of up to 18,000 IU/day of LMWH in obese patients with a BMI > 30 kg/m^2^ have been associated with a lower risk of recurrence, major bleeding, and all-cause death.^9^

### Cancer

In the setting of cancer-associated thrombosis, DOACs are now recommended over LMWH. However, apixaban or LMWH are preferred over other alternatives in patients with or at risk for gastrointestinal bleed due to malignancy.^5,17^ In a review by Lubetsky, the rate of VTE recurrence was lower or similar with DOACS compared to LMWH.^18^ The choice of treatment should take into account the presence of gastrointestinal malignancy, drug interactions, platelet levels, central nervous system metastasis, and patient preference.^7,9,18^

## Duration of Anticoagulation

Patients with PE should remain on anticoagulation for a treatment phase of 3 months as primary therapy.^5^ This recommendation includes patients with a transient provoking risk factor such as surgery with general anesthesia, confinement to bed for at least 3 days with an acute illness, Cesarean section, estrogen therapy, pregnancy or puerperium, trauma, leg injury associated with reduced mobility, and orthopedic or spinal injuries, to name a few.^5,19^ For pregnant women, anticoagulation should be continued during pregnancy, for at least 6 weeks after delivery, and extended for a treatment duration of 3 months if possible.^11^ In patients with an unprovoked PE or a PE associated with a persistent risk factor, anticoagulation should be extended over 3 months or until the risk factor has subsided, or it can be indefinite. Based on the risk of bleeding, anticoagulation can remain at full dose or an adjusted reduced dose provided that the therapeutic treatment advantage outweighs the potential risk of bleeding. Extended-phase anticoagulation that is not indefinite has been reported for a duration of 2 to 4 years.^5,20^

Certain guidelines recommend that all patients with PE receive 3 months or more of anticoagulation, citing that among patients who have had a PE, VTEs more frequently recur as a PE and the fatality rate with recurrence is twice as high as that of DVTs.^11^ The risk of PE recurrence has been reported as four-times higher in patients presenting with a symptomatic PE compared to patients with a proximal DVT.^20^ Studies have shown that all patients with idiopathic, unprovoked, or provoked VTE are at risk for recurrence. For surgery-related risk factors, the risk of recurrence has been reported at 0.7% per patient year compared with 4.2% to 4.5% for those with nonsurgical transient risk factors. The risk of recurrence is even higher in the setting of unprovoked VTE and may reach 7.4% per patient year. The cumulative incidence can range from 15% to 53% between 1 and 10 years. In addition, the incidence of VTE increases significantly after age 60, and men have a 3.6 relative risk of recurrence compared to women.^11,20^ Cancer patients have a heightened thrombotic risk due to the malignancy itself and the use of chemotherapy, with a reported 5% incidence of incidental PE.^21^ Therefore, extension of anticoagulation should be considered in at-risk patients.

Secondary or extended anticoagulation that considers risk factors for bleeding may include a full maintenance or a reduced dose. Apixaban was studied for the extended treatment of venous thromboembolism. In a double-blind study, 2,486 patients who had completed 6 to 12 months of anticoagulation were randomized to receive 2.5 mg or 5 mg twice a day and then compared with those on placebo. The extended anticoagulation for 12 months with apixaban at either dose reduced the risk of VTE recurrence without increasing the rate of major bleeding, which was low and similar to those on placebo. This study favors the use of the lower dose for extended prophylaxis treatment.^22^ A randomized double-blind study compared 10 mg or 20 mg of rivaroxaban with 100 mg of aspirin in patients who had also completed 6 to 12 months of anticoagulation and did not require extended treatment with therapeutic dosing. A total of 3,365 patients were included in this study and treated for 12 months. The risk of VTE recurrence was lower with rivaroxaban at either treatment dose compared to aspirin, without a significant increase in bleeding rates.^23^ These studies support the extended use of DOACs, suggesting the benefit of a reduced or lower prophylactic dosing regimen for the prevention of recurrent thrombosis.^5^

Aspirin has been suggested for patients with unprovoked PE who have already stopped anticoagulation.^5^ In one study, patients were considered eligible if they were diagnosed with a first-ever, unprovoked, proximal deep-vein thrombosis or PE and treated with VKAs for 6 to 18 months. A total of 403 patients were included and randomly assigned to aspirin 100 mg/day or placebo for 2 years of therapy. The primary outcome was the recurrence of DVT or pulmonary embolism. VTE occurred in 5.9% and 11.0% patients per year in the aspirin and placebo group, respectively.^24^ In patients with unprovoked VTE, aspirin therapy reduced the rate of recurrence by approximately 40% compared to placebo, without an increase in major bleeding at the studied dose.^25^ Aspirin in low doses offers an appealing, safe and cost-effective option for the long-term prevention of recurrent events in patients with unprovoked VTE.^26^

**Figure 1:**
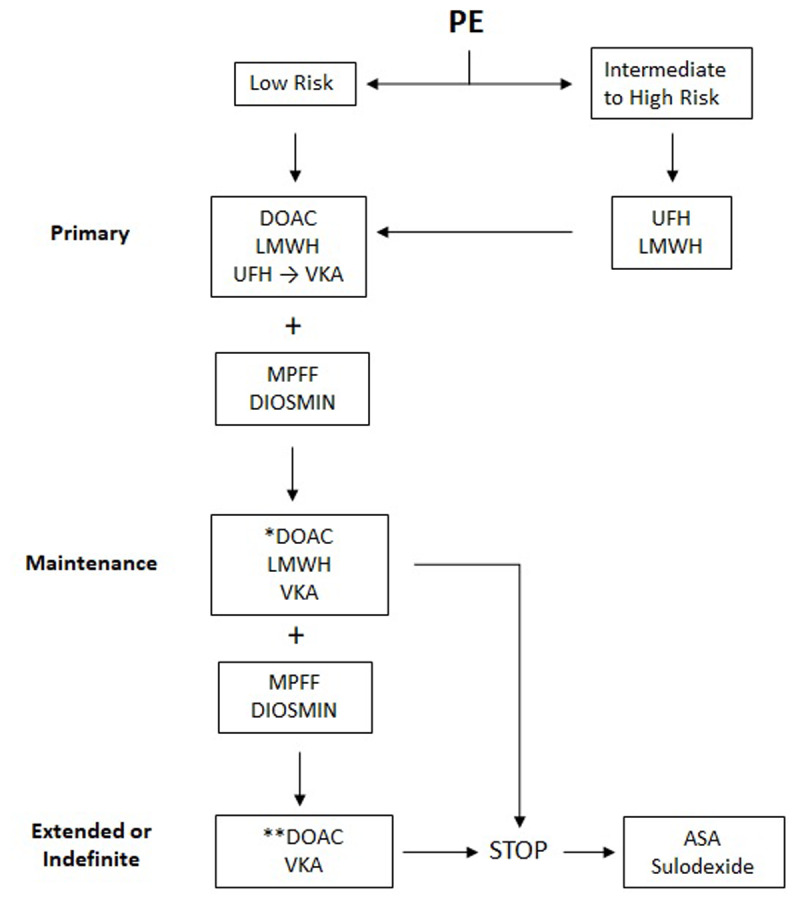
Algorithm summarizing the options of anticoagulation for patients with pulmonary embolism. PE: pulmonary embolism; DOAC: direct oral anticoagulant; LMWH: low-molecular-weight heparin; UFH: unfractionated heparin; VKA: vitamin K agonists; MPFF: micronized purified flavonoid fraction; ASA: acetylsalicylic acid * Full dose direct oral anticoagulants or reduced dose as per trials ** Usually at the reduced dose

Sulodexide is an oral mixture of glycosaminoglycans that exerts its antithrombotic and profibrinolytic effect by interacting with antithrombin III and heparin cofactor II and by inhibiting thrombin formation.^27,28^ Errichi and colleagues^29^ used sulodexide for the prevention of recurrent DVT in 405 patients who had completed treatment with anticoagulation for 6 months. The patients were separated into a treatment group and a control group. Sulodexide was then administered to the treatment group for a total of 24 months, with ultrasound scans repeated every 6 months. The incidence of recurrent DVT was significantly lower at multiple end points. In this multicenter registry, the incidence of recurrent DVT in the control group was 2.07 times higher than in the treatment group. In a multicenter double-blind study, 615 patients with a first-ever unprovoked venous thrombosis who had completed 3 to 12 months of oral anticoagulation treatment were randomly given sulodexide twice daily or placebo for 2 years in addition to compression stockings. Sulodexide given after discontinuation of anticoagulation significantly reduced the risk of DVT recurrence with no increase in bleeding risk.^27^ However, the reduction in recurrent PE was not significant in the small subset of patients between the groups in these two studies. These findings also are consistent with the previous trials on the use of aspirin for prevention. Based on these studies, it appears that sulodexide carries a lower hazard ratio for recurrence of VTE and relevant bleeding compared to aspirin.^30^ An algorithm summarizing the choice of anticoagulation is presented in Figure 1.

Evidence suggests that thrombophilia does not alter management. A family history of VTE or an unprovoked episode inherently suggests a higher patient risk. When testing is performed, it should be done off anticoagulation, and it often does not change the decision on duration of anticoagulation unless there is an antithrombin deficiency, combined thrombophilia, or acquired APS.^13^ No significant clinical benefit has been observed in extended anticoagulation treatment for carriers of heterozygous factor V Leiden or prothrombin 20210A mutation.^11^ The presence of elevated antiphospholipid antibodies with an associated VTE is reasonable indication for indefinite anticoagulation.^5,12,13^

Other factors should be considered when extending anticoagulation beyond the recommended initial 3 months. Since PE does not frequently occur in isolation and for the most part is associated with DVT, extension of anticoagulation should consider the underlying nidus of VTE and PE risk. For instance, a persistent chronic residual DVT may pose a risk factor for recurrence or extension of venous thrombosis if off anticoagulation. In addition, the duration of anticoagulation therapy should allow for the promotion of clot lysis and recanalization. In one study,^31^ 378 patients were randomized to receive either UFH and VKA or LMWH. Within 14 days, a 20% improvement in recanalization was confirmed by venography in the LMWH group versus those treated with UFH and VKA. At 84 days, recanalization was noted between 75.3% and 81.5% among both treatment groups. This study demonstrates that recanalization occurs over a treatment span that is initially more favorable with LMWH compared to UFH and VKA. In a study by Romera and colleagues,^32^ 241 patients with symptomatic DVT of the lower limbs were randomized to receive maintenance anticoagulation with LMWH or VKA. Venous recanalization assessed by duplex ultrasound increased significantly at 6 months (73.1% vs 47.5%) and at 12 months (91.5% vs 69.2%) with LMWH versus VKA. This demonstrates not only the efficacy of LMWH in achieving recanalization but also highlights how recanalization is a continuous process that still occurs at 6 and 12 months. In a retrospective analysis,^33^ a group of patients receiving UFH and VKA were compared with those on rivaroxaban. Recanalization at 30, 90, and 180 days for the UFH and VKA group was reported at 10%, 52.5%, and 78.9%, respectively. In contrast, recanalization was statistically more significant in the rivaroxaban group for the same time frame (55.3%, 83.5%, and 92.4%, respectively). Once again, the process of recanalization occurs beyond the 3-month primary treatment recommendation, and extension of therapeutic anticoagulation may benefit the recanalization process.

In a review by Robin and associates,^34^ residual pulmonary vascular obstruction was identified as a significant predictor of risk of recurrent VTE. Approximately, one-half of recurrences occurred within 1 year after discontinuation of anticoagulation therapy, corresponding to a risk of 8.1%. The 1-year risk of recurrent VTE was 5.8% in participants with < 5% residual pulmonary vascular obstruction compared to 11.7% in those with ≥ 5% residual obstruction. A significant decrease from baseline in D-dimer concentration of > 70% within the first month of anticoagulation has been associated with complete recanalization and decreased risk of recurrence after initial PE.^35^ However, the decrease in D-dimer concentration has been shown to continue at 6-month follow-up, and the decreased trend remained higher in the asymptomatic group compared to those with persistent symptoms, suggesting that recanalization or decreased coagulation activity persisted beyond the first month. These findings suggest that the ability to achieve recanalization beyond the 3-month primary anticoagulation phase has clinical implications for thrombus resolution, recanalization, and recurrence and should be factored into the decision to extend anticoagulation therapy (Table 2).

**Table 2:** Time segments for evaluation of anticoagulation therapy in patients with pulmonary embolism. PE: pulmonary embolism; ASA: aspirin; DOAC: direct oral anticoagulant; LMWH: low-molecular-weight heparin; UFH: unfractionated heparin; VKA: vitamin K agonists; ASA: acetylsalicylic acid


ANTICOAGULATION TREATMENT TIME SEGMENTS IN PE		

DURATION	CHOICE OF ANTICOAGULANT	COMMENTS

**Primary** 3 months	DOAC	Initial low risk patients and all post intervention

	LMWH	Parenteral anticoagulation with initiation of certain

	UFH/LMWH to VKA	DOACs and VKA

**Maintenance** 3-12 months	DOAC	Allows for continued fibrinolysis or recanalization

	LMWH	Supported by various trials at standard or reduced dose

	VKA	VKA for special populations

**Extended/lndefinite** 6-12 months - years	DOAC	Reduced DOAC dosing

	VKA	ASA or sulodexide when off anticoagulation

	ASA	Indefinite anticoagulation in special populations

	Sulodexide	


The implications of poor pulmonary artery recanalization are of significance. Chronic thromboembolic pulmonary hypertension (CTEPH) is caused by chronic thrombus and fibrous residue causing a mechanical obstruction of pulmonary arteries in PE survivors. Indefinite anticoagulation is recommended to prevent VTE recurrence. Other therapeutic approaches may include open pulmonary thromboendoarterectomy or percutaneous balloon pulmonary angioplasty.^13,36^ After PE, patients should be followed closely and assessed for a persistent, new-onset dyspnea or functional limitation. A diagnostic workup should be implemented to differentiate deconditioning, residual chronic thromboembolic disease, or CTEPH.^11^

## Adjuncts to Anticoagulation

Micronized purified flavonoid fraction (MPFF) is a compound that contains mostly diosmin and a small fraction of hesperidin. Micronization is a process that reduces the average diameter of solid particles to improve intestinal absorption. Both diosmin and hesperidin are naturally occurring flavonoid glycosides isolated from various plant sources.^37^ This venotonic class of compounds can serve as protective agents against the activation of inflammatory molecules, inhibit leukocyte adhesion, and have superoxide scavenger activities and an inhibitory effect against matrix metalloproteinases.^38,39^

An early study of 1,372 patients divided into various treatment categories showed that the combination of LMWH with Daflon (MPFF with added hesperidin) was more effective than LMWH alone in preventing postoperative symptomatic DVT and PE.^40^ Furthermore, the use of diosmin has proven effective in aiding recanalization in patients presenting with iliofemoral venous thrombosis. In one study, one group received initial LMWH followed by warfarin and diosmin, a second group received rivaroxaban only, and a third group received rivaroxaban with diosmin. The rivaroxaban-only group showed improved recanalization compared to the first group, whereas use of rivaroxaban with diosmin was more effective in achieving recanalization than the first two groups.^41^

A pilot clinical trial demonstrated that the use of MPFF together with rivaroxaban improved clinical outcomes in femoropopliteal DVT. Patients with confirmed femoropopliteal DVT by duplex ultrasound were randomized to a control group that received standard treatment with rivaroxaban or an experimental arm that received standard rivaroxaban treatment with the addition of MPFF for 6 months. At 6 months, the experimental group demonstrated a more rapid rate of recanalization and a reduced incidence of post-thrombotic syndrome.^42^ Similarly, the RIDILOTT DVT study^43^ randomized patients to receive standard treatment with rivaroxaban or with rivaroxaban and diosmin for 12 months. Adding diosmin was associated with a quicker and complete recanalization without a difference in DVT recurrence.

These studies demonstrate that the addition of venotonics such as MPFF or diosmin to anticoagulation aids in the process of recanalization of lower extremity DVT. It may be assumed that this improved recanalization will have a similar effect on a pulmonary artery thrombus, although further studies are needed to confirm this clinical inference. Figure 1 proposes an algorithm summarizing the options of anticoagulation in patients with PE.

## Conclusion

The preferential choice of anticoagulation management of PE includes DOACS. However, in special populations or selected patients, alternatives such as VKA or LMWH are preferred. Treatment beyond the 3-month primary phase should be considered, taking into account the risk of bleeding, underlying risk factors for recurrence, and even the benefit of thrombus resolution or recanalization. Using venotonic adjuncts with anticoagulation may help improve thrombus recanalization. Extended or indefinite anticoagulation should be considered in selected patients. Those who have stopped anticoagulation should be maintained on aspirin or sulodexide where the latter is available.

## Key Points

- Direct oral anticoagulants are the preferred outpatient treatment for anticoagulation both as full dose and reduced preventive dose.
- Special patient populations warrant titration or selection of alternate anticoagulation regimens.
- Duration anticoagulation should account for factors including risks of recurrence and recanalization.
- Venotonics may serve as an adjunct to anticoagulation in improving recanalization.
